# Ophthalmology Practice and Social Media Influences: A Patients Based Cross-Sectional Study among Social Media Users

**DOI:** 10.3390/ijerph192113911

**Published:** 2022-10-26

**Authors:** Hani B. ALBalawi, Osama Alraddadi

**Affiliations:** 1 Division of Ophthalmology, Department of Surgery, Faculty of Medicine, University of Tabuk, Tabuk 47512, Saudi Arabia; 2 Ohud Hospital, Ministry of Health, Medina 41412, Saudi Arabia

**Keywords:** social media, ophthalmologists, patients, Twitter, LinkedIn, Facebook, social networks, importance of social media, benefits of social media, practice

## Abstract

Many physicians consider social media a good tool for building their brands and attracting patients. However, limited data exist on patients’ perceptions of the value of social media in ophthalmology. Therefore, our objective was to examine how social media influences patients when choosing an ophthalmologist among social media users, and people’s behaviors toward ophthalmologists’ social media accounts. This was a cross-sectional study including 1086 participants. Males represented 77.3% of the sample. The majority of the participants (71.3%) were aged between 25 and 54 years. Regarding social media sites frequently checked, Twitter ranked first (75.3%), followed by Snapchat (52.8%) and YouTube (48.7%). The majority (92.3%) used social media sites at all times of the day. Concerning the importance of ophthalmologists’ social media sites, around 36.3% considered it either very or extremely important. As regards the important factors about an ophthalmologist’s social media site from participants’ perspectives, medical information written by the ophthalmologist (45.5%) and recommendations by friends (45.4%) were the most common reasons. Around 21% of females, compared to 16.8% of males, perceived the ophthalmologists’ social media sites as extremely important, *p* = 0.041. A quarter of participants aged between 18 and 24 years, compared to only 5.5% of those aged 65 and above, perceived the ophthalmologists’ social media sites as extremely important, *p* = 0.018. In conclusion, a considerable proportion of the people who used social media described ophthalmologists’ social media sites as very/extremely important in their choice of an ophthalmologist.

## 1. Introduction

As of 2019, there are 3.48 billion social media users worldwide [1], and the number is expected to increase. Initially, social media was used to connect with family membranes and old friends and follow celebrities, but now social media can also be used as a source of information, teaching, self-promotion, advertising, and others [2,3]. Almost all age groups and all community membranes are using social media nowadays. Parrish et al. indicated that adults aged 18 to 65+ use social media daily [4]. Almost 23% of the average time a user spends on the internet is on social media. Nathaniel & Adio point out that more than 80% of adults in the US use social media. Facebook has the largest share, and Americans spend more time on Facebook than any other site [5]. These figures have led more and more professionals (including Ophthalmologists) and small businesses to consider using the enormous power of social media [6]. Physicians are no exception, as one survey of more than 4000 medical doctors found that more than 90% of them are using some form of social media [7]. In the medical community, using social media creates unique opportunities to promote health, increase community awareness about some medical issues, and others. Despite these advantages, many concerns arise, such as the misuse of social media and the lack of regulations in many countries that leads to unprofessional personal online consultations, posts with false information, and other violations [8].

The importance of social media in ophthalmology has grown at a personal level, as many ophthalmologists use social media to build their brand—as do the upper levels of ophthalmology organizations, societies, and journals. A study published in 2015 noted that of 107 ophthalmology journals listed on SCImago, 21% had a Facebook account, and around 18% used Twitter [9]. At the individual level, according to Tsui and Rao, many Ophthalmologists are now using social media for professional communication with patients and colleagues [10]. Ting et al. stated that many ophthalmologists now turn social media into a form of communication and advertisement as well [11]. Moreover, Thanuskodi and Kumar stated that Ophthalmologists who want to participate in what is going on in the world and conversations with other ophthalmologists and patients should interact on social media [12]. Schmuter et al. also stated that doctors are no longer as isolated as before due to the use and implementation of social media in their daily practices [3]. Undoubtedly, potential patients have gradually come to see social media as an essential part of their daily lives.

Many factors may affect the patients’ decisions when choosing treating doctors—for example, years of experience, the reputation of the treating doctors, recommendations by relatives, and others. However, when searching the literature about the effect of social media on ophthalmology practice and, more specifically, on patients when selecting an ophthalmologist, any influences on patient decisions and determinations regarding the factors that attract patients to follow ophthalmologists’ social media accounts are missed. In fact, a deficit is also noticed in the literature regarding patients’ perspectives, in general, toward the ophthalmologist’s social media account. Other medical subspecialties, such as dermatology, share some similarities with ophthalmology services; for example, both contain elective procedures, i.e., not urgent, giving the patient the privilege of choosing the treating doctors. However, in contrast to ophthalmology, dermatology and the effects of social media were tested well, and many articles searched for the relationship that was missed in ophthalmology. For example, in a survey among social media users regarding choosing dermatologists in the USA, only 22% believed that social media was very/extremely important when choosing a dermatologist. Still, this same information was missing in reference to the field of ophthalmology [8].

Given the lack of research on the influence of social media on patients when choosing ophthalmologists among social media users and the lack of information related to people’s behaviors toward an ophthalmologist’s social media account content, the aims of this study were raised to examine how social media can influence patients who use social media when selecting an ophthalmologist. This article also discusses people’s behaviors toward ophthalmologist’s social media accounts and why and how ophthalmologists can use social media to build their brand based on patients’ views, as patients may view healthcare workers’ qualifications or comments provided by other patients or search for factors that do not necessarily represent physicians’ skills, such as issues related to their personal lives. We tested the hypothesis that social media may play an important part in patients’ decisions when selecting a treating ophthalmologist.

## 2. Materials and Methods

This cross-sectional study used self-administered electronic questionnaires via an online survey through SurveyMonkey^®^ (San Mateo, CA, USA) in December 2020. It was distributed randomly via social media to all reachable candidates.

Sample size:

The necessary sample size was determined using an online sample size calculator [13]. A confidence interval of 95%, a 5% error margin on a population of 30,000,000, and a response distribution of 50% were chosen. The representative sample size was 385 participants.

Data collection:

The questionnaire was obtained, with a few modifications, from previously published research with similar aims [8] and divided into five sections that included demographics (age, gender, marital status, occupation, residential area, and educational level); participants’ use preferences for social websites; typical social media use and behavior; and the relationships between choosing an ophthalmologist and their account, with a specific focus on the ophthalmologists’ accounts. In addition, two screening questions were used: “How often do you use social media?” and “Have you ever seen an ophthalmologist?” Those who use social media at any frequency and have seen an ophthalmologist were allowed to complete the survey.

A pilot sample of 10 participants was randomly selected to test the leading and complex questions; then, these ten subjects were excluded from the final analysis.

## 3. Results

The study included 1086 participants. Their demographic characteristics are presented in Table 1. Males represented 77.3% of the sample. The majority of the participants (71.3%) were aged between 25 and 54 years. More than half of the participants (53.7%) were Bachelor’s holders, whereas 16% were post-graduates. Most of the participants were recruited from Mecca (31%), Medina (26.1%), and Riyadh (14.1%).

**Figure 1:**
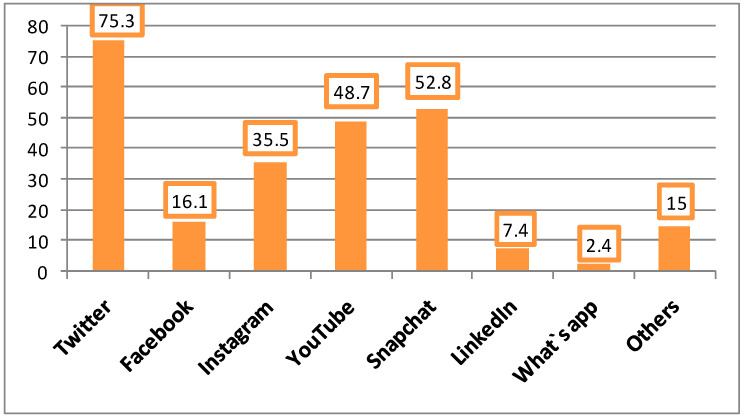
Social media sites frequently checked by the participants.

Regarding social media sites frequently checked by the participants, Twitter ranked first (75.3%), followed by Snapchat (52.8%), YouTube (48.7%), and Instagram (35.5%) Figure 1.

**Figure 2:**
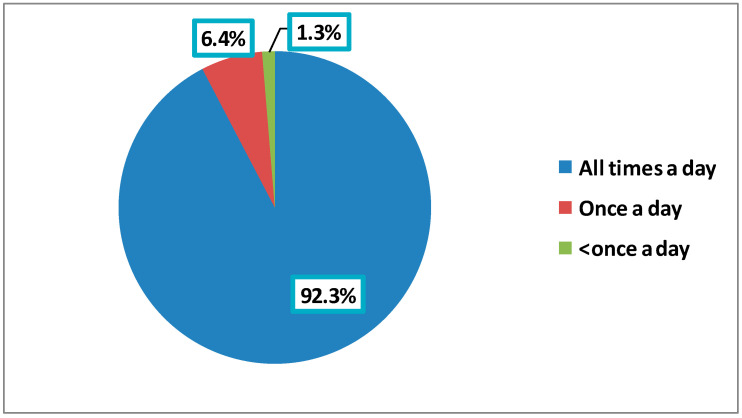
“How often do you use social media?”, as answered by the participants.

The vast majority of the participants (92.3%) used social media sites all the time, whereas only 6.4% used them once a day (Figure 2). Concerning the importance of an ophthalmologist’s social media site in patients’ decision to be seen by them, 26.9% of the participants considered it not at all important, whereas more than one-third of them (36.3%) considered it either very or extremely important. About half of the participants (49%) reported that the ophthalmologist met their expectations based on his or her social media site either mostly (33.8%) or completely (15.2%), while 23.6% described their expectations as either not at all (10.7%) or slightly (12.9%) being met (Table 2).

**Figure 3:**
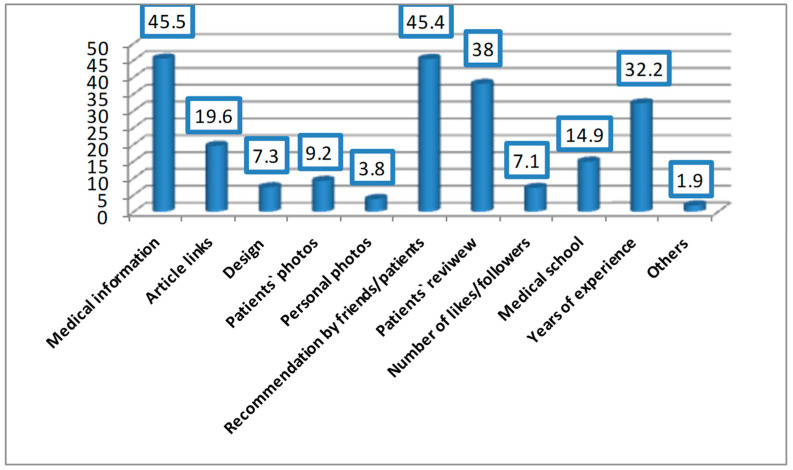
Important factors of an ophthalmologist’s social media site: Participants’ perspectives.

Regarding the important factors of an ophthalmologist’s social media site from participants’ perspectives, medical information written by the ophthalmologist (45.5%), recommendations by friends or patients (45.4%), and patient’s reviews about the ophthalmologist (38%) were the most valued (Figure 3).

Routine visits were the most frequently reported reasons for visiting an ophthalmologist (39.9%), followed by urgent cases (25.6%) and refractive surgery (19.1%), as seen in Table 3.

**Figure 4:**
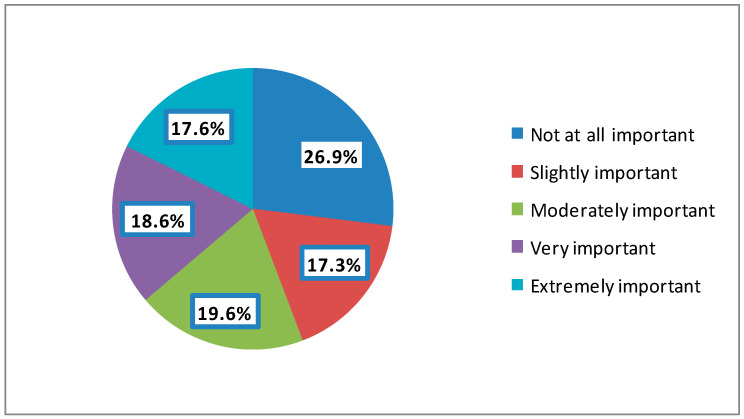
How important an ophthalmologist’s social media site is for patients.

More than one-fifth of females (21%), compared to 16.8% of males, perceived ophthalmologists’ social media sites as extremely important, *p* = 0.041. A quarter of participants aged between 18 and 24 years, compared to only 5.5% of those aged 65 and above, perceived ophthalmologists’ social media sites as extremely important, *p* = 0. 018. Almost a third (34.7%) of Master’s or Doctorate holders, compared to 21.4% of diploma holders, perceived ophthalmologists’ social media sites as not at all important, *p* = 0.011. Having a place of residence in the Kingdom of Saudi Arabia and the reasons for visiting an ophthalmologist were not associated with perceiving the importance of ophthalmologists’ social media sites (Table 4, Figure 4).

## 4. Discussion

The present study aimed to examine how social media can influence patients’ selection of an ophthalmologist and why and how ophthalmologists can use social media. Clearly, this survey showed that a large population uses social media daily, which provides ophthalmologists with a golden opportunity to use social media to attract users, send eye health messages, promote eye health, and increase awareness about eye issues. For example, people in Saudi Arabia showed a growing interest in using social media, which affects their daily lives. The number of social media applications has doubled during the last few years, from 8.5 million to 18.3 million users recently, which represents 58% of the Saudi people. Facebook (11 million users) and Twitter (9 million users) ranked first and second, followed by YouTube video clips (7 million) [14].

Additionally, among Arab countries, Saudi Arabia ranked first and worldwide, ranked second among WhatsApp and Snapchat users [14]. In the current survey, Twitter ranked first (75.3%), followed by Snapchat (52.8%), YouTube (48.7%), and Instagram (35.5%).

In this study, a routine visit was the most frequently reported reason for visiting the ophthalmologist, followed by urgent cases and refractive surgery. However, the decision to visit an ophthalmologist was not associated with patients’ perceptions of the importance of physicians’ social media sites in their choice.

In the present study, more than a third of the participants (36.3%) believed that physicians’ social media sites were very/extremely important when selecting an ophthalmologist. In another similar survey regarding dermatologists in the USA, only 22% believed that social media was very/extremely important when choosing a dermatologist [8]. The difference can be explained by the difference in the perceptions of patients in the USA and Saudi Arabia regarding the importance of social media in choosing a physician or can be explained by differences in selecting ophthalmologists as opposed to dermatologists, as ophthalmologists deal with more serious problems affecting vision. This result clearly showed that if ophthalmologists preferred to wait, they would be behind their colleagues and competitors, meaning that, unlike other colleagues, their names will not appear in any online search engine [15]. While this does not mean the social media-advertised practice is better, it is one of the factors that patients may be considering while choosing their doctors. Simply, these results suggest that it is important to build your online name and reputation. If a patient chooses between Dr. x and Dr. y, a better-looking social website with more than 5000 “likes” says you mean business.

In this study, the important factors that attracted the patients to an ophthalmologist’s social media site were the presence of medical information written by the ophthalmologist, recommendations by friends or patients, and patient reviews about the ophthalmologist. In another study, 71% of patients who utilized social media preferred to read educational material written by their physicians [16]. Further, Murphy et al. revealed that patients perceived the medical information written by the physicians themselves as the most attractive aspect of their social media sites [8]. It has been documented that written medical information should be simple enough to be understood by most people, including those who have lower levels of educational attainment [17,18]. This finding is of particular value in the present study, as lower-educated patients perceived ophthalmologists’ social media sites as extremely important—more than among higher-educated patients. The previously mentioned factors should be considered by ophthalmologists when using social media if they want to attract followers and start building up their trade name on social media.

Creating a social website account is cost-free, and it is the first and easiest step. However, many expert ophthalmologists in the field offer much advice, such as keeping the account very dynamic and frequently updated; posting about international days of awareness, such as world sight day; and using simple and straightforward language. On the other hand, despite online posting providing an excellent, efficient means of contact with many people, it carries certain risks for physicians that other businesses do not face. It is better to avoid pointless arguments, refrain from diagnosing any disease through social media, and abstain from providing medical advice without a clinical examination. However, the most important issue is to maintain the confidentiality of patients and not post any names, pictures, or any other items that may lead to the identification of patients without patient permission, and it is important to always refer to the Health Insurance Portability and Accountability Act HIPAA [15] Table 5.

Among the important limitations of this study is that we could not compare our findings with others, as searching online did not yield any similar studies regarding patients’ perceptions towards ophthalmologists’ social media sites, and only limited studies were carried out concerning patients’ perceptions of dermatologists. Furthermore, the cross-sectional design is another limitation, as it proves association rather than causality. Another significant limitation is the online approach utilized to collect the data in this study. The participants did not represent the general population; thus, the study was subject to selection bias. However, we investigated the impact of social media sites of ophthalmologists; therefore, it is appropriate to select only social media users.

This study filled the gap in the literature about the effect of social media on ophthalmology practice and answered the most controversial questions, such as social media’s influences on patient decisions. In addition, it provided background pieces of advice for ophthalmologists when professionally using social media.

## 5. Conclusions

Doctors are no longer as isolated as before due to the use and implementation of social media in their daily practices. Now, patients can use social media to find different medical information, treating doctors, and others. A considerable proportion of the people described ophthalmologists’ social media sites as very/extremely important in their choice of an ophthalmologist. Therefore, we recommended encouraging ophthalmologists to add simple, useful information on their social media sites for their patients and to take care of these sites, as they are perceived by a considerable proportion of patients to be important in their decision to select an ophthalmologist. In addition, further study is recommended to evaluate whether people remember their doctors and analyze their websites and uses of social networks.

## Figures and Tables

**Table 1:** Demographic characteristics of the participants (*n* = 1086).

	Valid Number (Total Participants)	Frequency	Percentage
Gender	1077		
Male		833	77.3%
Female		244	22.7%
Age (years)	1082		
18–24		72	6.7%
25–34		227	21.0%
35–44		282	26.0%
45–54		263	24.3%
55–64		182	16.8%
≥65		56	5.2%
Highest qualification/degree	1083		
High school		196	18.1%
Diploma		132	12.2%
Bachelor’s		581	53.7%
Master’s		125	11.5%
Doctorate/Ph.D.		49	4.5%
Place of residence	1082		
Riyadh		153	14.1%
Mecca		335	31.0%
Eastern province		93	8.6%
Medina		282	26.1%
Hail		56	5.2%
Jazan		53	4.9%
Qassim		39	3.6%
Asir		18	1.7%
Tabuk		21	1.9%
Jawf		16	1.5%
Others		16	1.5%

**Table 2:** Descriptive details of using social media sites among the participants.

	Valid Number	Frequency	Percentage
Frequency of using social media	1084		
All times of day		1001	92.3
Once a day		69	6.4
<Once a day		14	1.3
Importance of an ophthalmologist’s social media site in a patient’s decision to be seen by them	1080		
Not at all important		290	26.9
Slightly important		186	17.2
Moderately important		212	19.6
Very important		201	18.6
Extremely important		191	17.7
Did the ophthalmologist meet patients’ expectations based on his or her social media site?	792		
Not at all		85	10.7
Slightly		102	12.9
Somewhat		217	27.4
Mostly		268	33.8
Completely		120	15.2

**Table 3:** Reasons for visiting ophthalmologists among the participants (*n* = 1080).

	Frequency	Percentage
Refractive surgery	206	19.1
Routine visit	432	39.9
Follow-up visit	109	10.1
Urgent case	276	25.6
Never visit	54	5.3

**Table 4:** Factors associated with perceptions of the importance of ophthalmologists’ social media sites among the participants.

	Perceived Importance of Ophthalmologist’s Social Media Sites					*p*-Value
	Not at All Important	Slightly Important	Moderately Important	Very Important	Extremely Important	
	*N* = 255	*N* = 177	*N* = 209	*N* = 195	*N* = 185	
	*N* (%)	*N* (%)	*N* (%)	*N* (%)	*N* (%)	
Visit reason (*n* = 1021)						
Refractive surgery (*n* = 206)	45 (21.8%)	35 (17.0%)	40 (19.4%)	40 (19.4%)	46 (22.3%)	
Routine visit (*n* = 431)	118 (27.4%)	74 (17.2%)	86 (20.0%)	83 (19.3%)	70 (16.2%)	
Follow-up visit (*n* = 108)	25 (23.1%)	15 (13.9%)	21 (19.4%)	25 (23.1%)	22 (20.4%)	
Urgent case (*n* = 276)	67 (24.3%)	53 (19.2%)	62 (22.5%)	47 (17.0%)	47 (17.0%)	0.705
Gender (*n* = 1072)						
Male (*n* = 829)	230 (27.7%)	153 (18.5%)	155 (18.7%)	152 (18.3%)	139 (16.8%)	
Female (*n* = 243)	59 (24.3%)	32 (13.2%)	54 (22.2%)	47 (19.3%)	51 (21.0%)	0.041
Age (years)(*n* = 1077)						
18–24 (*n* = 72)	18 (25.0%)	11 (15.3%)	13 (18.1%)	12 (16.7%)	18 (25.0%)	
25–34 (*n* = 226)	68 (30.1%)	31 (13.7%)	48 (21.2%)	35 (15.5%)	44 (19.5%)	
35–44 (*n* = 281)	77 (27.4%)	55 (19.6%)	55 (19.6%)	38 (13.5%)	56 (19.9%)	
45–54 (*n* = 262)	55 (21.0%)	49 (18.7%)	55 (21.0%)	59 (22.5%)	44 (16.8%)	
55–64 (*n* = 181)	51 (28.2%)	31 (17.1%)	34 (18.8%)	39 (21.5%)	26 (14.4%)	
≥65 (*n* = 55)	21 (38%)	8 (14.5%)	6 (10.9%)	17 (30.9%)	3 (5.5%)	0.018
Highest qualification/degree (*n* = 1078)						
High school (*n* = 193)	64 (33.2%)	25 (13.0%)	36 (18.7%)	28 (14.5%)	40 (20.7%)	
Diploma (*n* = 131)	28 (21.4%)	20 (15.3%)	26 (19.8%)	25 (19.1%)	32 (24.4%)	
Bachelor’s (*n* = 581)	138 (23.8%)	107 (18.4%)	121 (20.8%)	115 (19.8%)	100 (17.2%)	
Master’s (*n* = 124)	43 (34.7%)	24 (19.4%)	24 (19.4%)	19 (15.3%)	14 (11.3%)	
Doctorate/Ph.D. (*n* = 49)	17 (34.7%)	9 (18.4%)	4 (8.2%)	14 (28.6%)	5 (10.2%)	0.011
Place of residence						
Riyadh (*n* = 153)	42 (27.5%)	30 (19.6%)	24 (15.7%)	30 (19.6%)	27 (17.6%)	
Mecca (*n* = 333)	97 (29.1%)	58 (17.4%)	68 (20.4%)	58 (17.4%)	52 (15.6%)	
Eastern province (*n* = 93)	21 (22.6%)	20 (21.5%)	22 (23.7%)	13 (14.0%)	17 (18.3%)	
Medina (*n* = 280)	72 (25.7%)	48 (17.1%)	55 (19.6%)	59 (21.2%)	46 (16.4%)	
Hail (*n* = 56)	12 (21.4%)	8 (14.3%)	9 (16.1%)	9 (16.1%)	18 (32.1%)	
Jazan (*n* = 53)	15 (28.3%)	6 (11.3%)	11 (20.8%)	11 (20.8%)	10 (18.9%)	
Qassim (*n* = 38)	10 (26.3%)	7 (18.4%)	6 (15.8%)	7 (18.4%)	8 (21.1%)	
Asir (*n* = 18)	7 (38.9%)	2 (11.1%)	4 (22.2%)	2 (11.1%)	3 (16.7%)	
Tabuk (*n* = 21)	4 (19.0%)	2 (9.5%)	5 (23.8%)	4 (19.0%)	6 (28.6%)	
Jawf (*n* = 16)	4 (25.0%)	3 (18.8%)	3 (18.8%)	5 (31.3%)	1 (6.3%)	
Others (*n* = 16)	5 (31.3%)	2 (12.5%)	4 (25.0%)	2 (12.5%)	3 (18.8%)	0.948

**Table 5:** Tips when using social media.

Better to Do	Better to Avoid
Keep the account very dynamic and frequently updated	Pointless arguments
Post about international eye awareness days	Diagnosing diseases through social media, or writing any medical advice without a clinical examination
Use simple and straightforward language	Posting names, pictures, or any other items that may lead to the identification of patients
Maintain the confidentiality of patients	

## Data Availability

All the data presented in this manuscript are available upon request.
